# Is Open-angle Glaucoma Caused by the NO/ONOO(-) Cycle Acting at Two Locations in the Eye?

**Published:** 2014

**Authors:** Martin L Pall

**Affiliations:** Professor Emeritus of Biochemistry and Basic Medical Sciences, Washington State University, USA

**Keywords:** Open-angle Glaucoma; NO/ONOO(-) Cycle; NMDA Receptors; Trabecular Meshwork; Nrf2

The NO/ONOO(-) cycle is primarily a local complex, biochemical, vicious cycle mechanism based on well-established processes (1). The cycle is thought to be the possible cause of different diseases when localized in various body tissues. The most extensively documented of these is heart failure, where the impact of the cycle in the myocardium causes the vast changes that occur in heart failure (1). The main question being raised here is whether the impact of the NO/ONOO(-) cycle in the trabecular meshwork, and subsequently in the retinal ganglion, is the cause of open-angle glaucoma. An important notion here is that the high intraocular pressure (IOP) resulting from changes in the trabecular meshwork produces physical trauma and elevated NMDA activity; there is consequent initiation of the NO/ONOO(-) cycle in the retinal ganglion, leading to retinal ganglion cell (RGC) degeneration. A second question relates to whether the high IOP in closed-angle glaucoma, and also highly variable but normal range IOP in other types of glaucoma, may both act on the retinal ganglion via physical trauma and the NO/ONOO(-) cycle to produce RGC degeneration.

The NO/ONOO(-) cycle has 12 different elements which are increased by the cycle in the impacted tissue (1). These elements include: nitric oxide (NO), superoxide, peroxynitrite, oxidative stress, NF-kB, inflammatory cytokines, iNOS induction, mitochondrial dysfunction, excitotoxicity including excessive NMDA activity, intracellular calcium, elevated activity of several of the TRP receptors, and tetrahydrobiopterin depletion. All except the last two of these have been examined and shown at increased levels in the retinal ganglion in glaucoma. Most of these elements have been explored and also found at elevated levels in the trabecular meshwork in open-angle glaucoma.

Let’s consider the trabecular meshwork first. One of the stressors that produces the trabecular remodeling found in open-angle glaucoma is H(2)O(2) treatment. Li et al. (2) found that H(2)O(2) treatment of the trabecular meshwork produced chronic elevation of oxidative stress, as well as that of NO, iNOS, NF-kB, inflammatory cytokines and mitochondrial dysfunction—six elements of the NO/ONOO(-) cycle. Other studies have implicated both peroxynitrite and intracellular calcium, in addition to the aforementioned six cycle elements, in glaucoma-associated trabecular meshwork changes. Still other changes linked to the NO/ONOO(-) cycle in heart failure (1)—matrix metalloproteinase (MMP), calpain and endothelin-1 elevation—are also implicated in the trabecular meshwork changes in open-angle glaucoma. The finding that the oxidant H(2)O(2) treatment generates chronic changes, including oxidative stress, strongly suggests a vicious cycle in the generation of the trabecular meshwork changes. The finding of elevation of so many NO/ONOO(-) cycle elements in this trabecular meshwork process strongly suggests that the NO/ONOO(-) cycle is the vicious cycle involved. MMP elevation and many other changes found in the tissue remodeling in heart failure can all be causally linked to NO/ONOO(-) cycle elements (1), suggesting that remodeling of the trabecular meshwork in glaucoma may be similarly explained.

Physical trauma has been shown to produce excessive activity of the NMDA receptors in the brain and spinal cord, subsequently leading to traumatic brain injury and spinal cord injury. In various publications the author has discussed how excessive NMDA activity may be involved in initiating cases of diseases apparently caused by the NO/ONOO(-) cycle. The role of excessive NMDA activity in causing RGC degeneration in glaucoma has been reviewed by Seki and Lipton (3). It is reasonable, therefore, that high or highly variable IOP creates physical trauma on the RGCs in the region where the retinal ganglion leaves the eye, thereby producing excessive NMDA activity via several possible mechanisms, as discussed earlier (3). The excessive NMDA activity may act on the RGCs to initiate the NO/ONOO(-) cycle in the retinal ganglion, causing glaucoma.

Most of the NO/ONOO(-) cycle elements have been studied and shown to be raised in the retinal ganglion in glaucoma, namely: superoxide, nitric oxide, peroxynitrite, oxidative stress, NF-kB, inflammatory cytokines, iNOS, mitochondrial dysfunction, NMDA activity and intracellular calcium (4,5) with most of these having causal roles in RGC changes and apoptosis. This pattern of evidence makes it highly likely that the NO/ONOO(-) cycle has a central causal role in the retinal ganglion in glaucoma. Because the NO/ONOO(-) cycle produces mitochondrial changes that can lead to apoptosis (1), its apparent role here makes mechanistic sense.

It is the author’s view that treatments raising Nrf2 activity are likely to be promising treatments for glaucoma because of the role of Nrf2 in lowering multiple NO/ONOO(-) cycle elements. This position is also suggested by Miyamoto et al.’s recent study of quercetin acting by raising Nrf2 in glaucoma treatment.

In summary, the local impact of the NO/ONOO(-) cycle in the trabecular meshwork may lead to remodeling of that meshwork and consequent high IOP. Other mechanisms in closed-angle and other types of glaucoma can also lead to high or variable normal range IOP, both of which act via physical trauma on RGC to elevate NMDA activity and initiate the NO/ONOO(-) cycle in RGC. The NO/ONOO(-) cycle in the RGC produces both apoptotic cell death and other changes, including tissue remodeling. Agents that raise Nrf2 may thus be expected to be effective in glaucoma treatment however more studies are required. 

On behalf of editorial board, it is our pleasure to announce that PubMed central has accepted the journal, “Med Hypothesis Discov Innov Ophthalmol” for inclusion in its database. This means that the articles published in our journal will now be available to search using PubMed, which most practitioners use to access academic resources. We have seen not only a pleasing increase in unsolicited submissions to the journal but also increased readership over these last few months. In this issue most articles are related to glaucoma and we hope you enjoyed reading the Journal content.
